# Light intensity and cage position affect meat quality by regulating intestinal flora, inflammation and oxidation in broilers

**DOI:** 10.3389/fmicb.2026.1729385

**Published:** 2026-02-25

**Authors:** Fei Zhang, Zhentiao Gao, Fang Wang, Yuyang Xue, Shanshan Nan, Wei Jing, Yifan Nie, Tianyu Lu, Xueqiang Liu, Cunxi Nie

**Affiliations:** 1 College of Animal Science and Technology, Shihezi University, Shihezi, China; 2 Xinjiang Chuangyu Poultry Farming Co., Ltd, Shihezi, China

**Keywords:** cage position, gut microbiota, inflammation, light intensity, meat quality, oxidation, yellow-feathered broiler

## Abstract

**Introduction:**

With improvements in living standards, high-quality food has become the preferred choice for high-quality living, leading to an increasing demand for chicken meat quality. Light intensity and cage position are crucial environmental factors in intensive farming systems and serve as environmental stressors linked to systemic inflammation and oxidative status in broilers. This study investigated the effects of light intensity and cage position on Jinling yellow-feather broiler meat quality.

**Methods:**

A total of 1,200 male yellow-feathered broilers were randomly assigned to a 2 × 3 factorial arrangement. Treatments included two light intensities (4 lx and 1.5 lx) and three cage positions (upper, middle, and lower). There were six treatment groups: 4 lx–upper (Ht), 4 lx–middle (Hm), 4 lx–lower (Hs), 1.5 lx–upper (Lt), 1.5 lx–middle (Lm), and 1.5 lx–lower (Ls). Each group contained 10 replicates (cages) with 20 broilers each.

**Results:**

The 4 lx treatment significantly improved pH, and meat color (*p* < 0.05) while reducing cooking loss and drip loss (*p* < 0.05). Breast muscle and leg muscle pH_24h_ in the middle showed significantly higher than the upper (*p* < 0.05). Significant light × cage position interactions affected breast muscle pH_24h_, meat color, protein content, IMF, Tyr, and C16:0 (*p* < 0.05); leg muscle pH_45min_, pH_24h_, cooking loss, Glu, and SFA (*p* < 0.05). Briefly, the Ht and Hm groups had higher levels of meat quality. In terms of serum inflammatory status and oxidative levels, with the exception of GSH-Px, all indicators were significantly influenced by the interaction of light intensity × cage position. The Hm group had significantly higher IL-10 levels and total antioxidant capacity, indicating reduced systemic inflammation and enhanced antioxidant capacity. Furthermore, *Alistipes* and *Barnesiella* were significantly enriched in the Hm group, negatively correlated with cooking loss in the breast muscle, and positively correlated with T-AOC. *Faecalibacterium, unclassified_f_Lachnospiraceae*, and *Ruminococcus_torques_group* were negatively correlated with drip loss in both breast and leg muscles and positively with IL-10.

**Conclusions:**

These findings suggest that light intensity and cage position interact to affect meat quality, with observed improvements potentially linked to concomitant changes in gut microbiota, inflammation and oxidative status.

## 1 Introduction

With economic development boosting consumer purchasing power, high-quality food has become an essential component of high-standard living, leading to increasingly stringent requirements for chicken meat quality (Suo et al., 2016). Against the backdrop of the rapid expansion of China's intensive broiler industry, multi-tier cage systems have emerged as the dominant rearing model owing to their advantages of high space utilization efficiency, enhanced production efficiency, and reduced feed waste.

Meat quality is a critical factor that influences consumer purchase decisions (Choi et al., 2023). Previous studies have indicated that, in addition to genetics, nutrition, and processing methods, poultry housing and management conditions significantly affect chicken meat quality (Baéza et al., 2022). For instance, Sakamoto et al. (2024) compared positive and negative pressure ventilation systems in chicken houses and found that negative pressure ventilation reduced heat stress and improved meat quality by increasing muscle pH Wang et al. (2022) investigated the effects of broiler stocking density and observed higher fat content in leg muscles under low- and medium-density conditions, suggesting a potential correlation between certain microbiota (such as *Faecalibacterium*) and fat deposition Son et al. (2022) studied the impact of stocking density under high-temperature conditions and demonstrated that a lower density alleviated heat-induced oxidative stress, thereby improving meat quality by reducing cooking loss and shear force. Research has established that key indicators of meat quality include pH, color, and tenderness, and studies within the past 5 years have highlighted growing interest in strategies to enhance chicken meat quality (Deng et al., 2024; Zhou et al., 2024). However, the interactive effects of light intensity and cage position in multi-tier cage systems on broiler meat quality remain unclear.

We hypothesized that light intensity and Cage position might regulate meat quality in broilers by modulating the gut microbiota structure. To test this hypothesis, Jinling yellow-feathered broilers were used as model organisms. We systematically evaluated the effects of different light intensities and cage tiers on the meat quality, serum inflammatory cytokine levels, oxidative stress markers, and cecal microbiota composition. The aim of this study was to elucidate the impact of the light environment and cage position on meat quality in Jinling yellow-feathered broilers and to explore the underlying mechanisms.

## 2 Materials and methods

### 2.1 Experimental design

The experiment followed a 2 x 3 factorial design to investigate the effects of light intensity and cage position. The first factor, light intensity, comprised two levels: 4 lx and 1.5 lx, achieved by central regulation of the poultry house illumination. The second factor was the cage position, comprising three levels: Upper, Middle, and Lower cage position.

A total of 1,200 1 day-old male yellow-feathered broilers were randomly assigned to six treatment groups. The cage was defined as the experimental unit. Each treatment group consisted of 10 replicate experimental units (a total of 60 cages), with 20 broilers per cage (cage dimensions: 1.2 m × 1.0 m × 0.9 m). The experimental groups are listed in Table 1.

**Table 1:** Experimental design and grouping.

**Light intensity (lx)**	**Cage position**		
	**Upper (t)**	**Middle (m)**	**Lower (s)**
4 lx (H)	4 lx-upper (Ht)	4 lx-middle (Hm)	4 lx -lower (Hs)
1.5 lx (L)	1.5 lx-upper (Lt)	1.5 lx-middle (Lm)	1.5 lx-lower (Ls)

The test set the light factor as the average light intensity in the whole enclosure, and the cage layer factor conclude the actual light, wind speed, temperature and other complex factors caused by the difference in location.

### 2.2 Experimental animals and management

The experiment was conducted at Xinjiang Chuangyu Poultry Farming Co., Ltd. Using a three-tier cage system, the experimental broilers were housed in the upper, middle, and lower cage positions within two poultry houses. During the test period, LED lights were used as the light source, and constant illumination was maintained at a cycle of 19L:5D (Light: 00:00-19:00; Dark: 19:00-24:00, Beijing Time). The mean light intensities were maintained at 4 lx (upper: 4.7 lx; middle: 4.0 lx; lower: 3.3 lx) in Poultry House 1 and 1.5 lx (upper: 1.8 lx; middle: 1.5 lx; lower: 1.2 lx) in Poultry House 2, measured using a TES-1332A digital illuminance meter (Taiwan, China). Prior to the experiment, other environmental parameters were measured (measurements were standardized by inserting the probe horizontally approximately 10 cm into the cage through the door at each tier). The results indicated that the temperature and air velocity were consistent between the two poultry houses (temperature: 23 ± 0.5 °C; air velocity: 2.5 ± 0.3 m/s).

From 1 to 21 days of age, the chicks were reared according to conventional feeding standards and immunization protocols. The experimental period commenced at 22 days of age and continued until 68 days of age, spanning 46 days. Throughout the test cycle, four times a day to feed, chickens were provided with free access to feed and water (Drinking water is provided through a water dispenser), and managed according to standard immunization procedures. The chicken coop was equipped with an exhaust fan for ventilation, and feces boards were cleaned of fecal matter every day. The basal diet was formulated according to the Chinese Agricultural Standard Nutrient Requirements for Yellow-feathered Broilers (NY/T 3645-2020) and prepared by Xinjiang Chuangyu Poultry Farming Co., Ltd. The dietary composition and nutrient levels are presented in Table 2.

**Table 2:** Base diet composition and nutrient content.

**Items**	**22-42 d**	**43-68 d**
**Ingredients, %DM**		
Corn	50	56
Soybean meal (48% crude protein)	9.5	17
Cottonseed protein (50% crude protein)	9	7.5
Corn protein powder (60% crude protein)	5	-
Maize	10	6
Soybean oil	5	4
Multi-dimensional and multi-mineral premix^a^	7	5
L-Methionine	0.2	1
Lysine	0.3	-
Calcium biphosphate	2	-
Threonine	-	0.25
Tryptophan	-	0.25
Shellac	0.8	2
Common salt	1.2	1
Add up the total	100	100
**Nutritional content** ^b^		
Metabolizable energy, MJ/kg	12.45	12.39
Crude protein, %	18.56	18.63
Crude fat, %	2.68	2.19
Calcium, %	0.97	0.97
Phosphorus, %	0.69	0.67

^a^ Each kg of premix contains: VA 11550 IU, VD 4200 IU, VE 28 IU, VK 28 mg, VB1 2.1 mg, VB2 8.75 mg, VB6 3.5 mg, VB12 0.021 mg, D-Biotin 0.105 mg, VB9 1.4 mg, Niacin 42 mg, D-Pantothenic acid 11.2 mg, Fe 100 mg, Cu 8 mg, Mn 120 mg, Zn 80 mg, Se 0.3 mg, I 0.7 mg, Co 0.5 mg. 11.2 mg, Fe 100 mg, Cu 8 mg, Mn 120 mg, Zn 80 mg, Se 0.3 mg, I 0.7 mg, and Co 0.5 mg.

^b^ Metabolizable energy is calculated, and the rest of the nutrient levels are measured.

### 2.3 Sample collection

At 68 days, after a 12h fast, 10 broilers were randomly selected from each treatment group (one bird from each replicate cage) were randomly selected from each group for venous blood collection. To induce loss of consciousness and death with a minimum of pain and distress, broilers were euthanized by an intravenous injection of 5% sodium pentobarbital was performed according to the method of (Liu et al., 2021). Once a surgical plane of anesthesia was confirmed by the loss of pedal reflex, exsanguination via severing the jugular veins was ensure death. After post-slaughter, samples of major and leg muscle were removed. The contents of the cecum was squeezed out into a 2 mL lyophilizer tube, flash frozen in liquid nitrogen, and stored (−80 °C) for later analysis.

To ensure sufficient biological redundancy, 10 broilers were initially sampled per group. From this pool, seven samples were selected for serum analysis after excluding hemolyzed specimens. Subsequently, to ensure data consistency, samples for further analysis and assays were from the individuals corresponding to the seven non-hemolyzed serum samples.

### 2.4 Muscle appearance indicators

After ipsilateral pectoralis major and leg muscles were excised. Identical cross-shaped markings were applied at standardized locations in both muscle groups. Meat color parameters were quantified using a chroma meter (OPTO-STAR, Germany), and the pH was measured using a portable pH meter (pH 3110 SET 2, Germany) at 45 min and 24 h post-mortem. One hour after slaughter, whole muscle samples (minimum dimensions: 6 × 3 × 3 cm) were excised. Superficial connective tissue and fat were trimmed, after which samples were vacuum-sealed and refrigerated at 4 °C. After 24 h, samples were removed, equilibrated to room temperature (25 °C), and weighed to record initial mass (W_1_). Subsequently, samples were heated in a precision water bath at 80 °C for 30 min, cooled to 0–4 °C, blot-dried with filter paper, and reweighed (W_2_). Cooking loss was calculated using the formula:


$$Cooking\;loss\;(\%)=\frac{\left(W1-W2\right)}{W1}\times 100$$

Following cooking loss determination, samples were trimmed into 1 cm × 1 cm cross-sections, and shear force (N) was measured using a meat tenderness tester (MAQC-12, China), with samples oriented perpendicular to the muscle fibers within the blade fixture. Measurements were performed in triplicate per sample, and the mean values were calculated. A separate meat sample was excised parallel to the orientation of the muscle fibers. Pre-storage weight (W_3_) was recorded, after which the sample was suspended by a stainless-steel hook in a sealed bag and stored vertically at 4 °C. After 24 h, samples were removed, surface moisture was gently blotted, and reweighed (W_4_). Drip loss was calculated as follows:


$$Drip\;loss\;(\%)=\frac{\left(W3-W4\right)}{W3}\times 100$$

### 2.5 Muscle nutritional composition

Following refrigeration at 4 °C, meat samples were processed by trimming superficial fat and connective tissue, and then homogenized through mincing. Precisely 80 g ± 0.5 g aliquots of minced meat were uniformly distributed in 11 cm Petri dishes and analyzed for intramuscular fat (IMF) and protein content using a near-infrared food analyzer (MultiScan 5,000, Australia; spectral range 900-1,700 nm) calibrated with NIST-traceable standards.

### 2.6 Muscle fatty acid composition analysis

Fatty acid composition was determined following Zhang et al. (2025) with modifications: 5 g aliquots of fresh muscle were freeze-dried for 48 h (LGJ-10 Freeze Dryer, China) and pulverized into lyophilized powder for subsequent analysis. Lipids from 0.5 g lyophilized muscle underwent base-catalyzed methylation using 4 mL 0.5 mol/L NaOH-CH_3_OH, followed by acid-mediated extraction with 4 mL 2% H_2_SO_4_-CH_3_OH. Fatty acid methyl esters (FAME) were then partitioned into the organic phase by addition of 2 mL n-hexane and 2 mL distilled water. The mixture was vortexed (5 min) and centrifuged (3,500 x g, 5 min). The FAME-containing organic phase was transferred to a new tube with 0.25 g activated charcoal and 0.25 g anhydrous Na_2_SO_4_, shaken until decolorized, and stored overnight. Post-incubation, the supernatant was re-centrifuged (3,500 rpm, 5 min), further clarified (13,000 rpm, 5 min), and filtered through a 0.45 μm PTFE membrane into GC vials. The fatty acids (FA) were determined using a gas chromatography-triple quadrupole mass spectrometer (Agilent 8,890-7,000 DGC-MS, USA). Injection volume was 10 μL with the following temperature program: initial hold at 80 °C for 3 min, increased to 195 °C at 6 °C/min (held for 2 min), then to 230 °C at 1 °C/min. The detector was operated isothermally at 250 °C throughout the analysis. Fatty acid profiles were identified using a 37-component FAME mix standard (AccuStandard), with sample peaks assigned by retention time matching (±0.05 min tolerance). Sample peaks were identified based on the retention time, and the concentration of individual FA was determined from the peak areas of known standards.

### 2.7 Muscle amino acid composition analysis

Fresh meat samples (0.2000 ± 0.001 g) were hydrolyzed with 15 mL 6 mol/L HCl in a hydrolysis tube at 110 °C for 22 h. The hydrolysate was filtered through quantitative filter paper and diluted to 50 mL in a volumetric flask. A 1.0 mL aliquot was dried under reduced pressure (RE-52AA rotary evaporator, China; 40 °C-50 °C), rehydrated in deionized water, and re-evaporated to complete dryness. The residue was dissolved in 2 mL sodium citrate buffer (pH 2.2), centrifuged at 13,000 × g (10 min), and filtered through a 0.45 μm PTFE membrane into GC vials for analysis. Muscle tissue amino acid profiles were quantified using an amino acid analyzer (Hitachi L-8900, Japan), detecting the following 16 compounds: Aspartic (Asp), Threonine (Thr), Serine (Ser), Glutamic (Glu), Glycine (Gly), Alanine (Ala), Valine (Val), Methionine (Met), Isoleucine (Ile), Leucine (Leu), Tyrosine (Tyr), Phenylalanine (Phe), Lysine (Lys), Histidine (His), Arginine (Arg), and Proline (Pro). Flavor amino acids were classified according to the sensory evaluation criteria described by Luo et al. (2022).

### 2.8 Serum inflammatory status and oxidative levels

Blood samples were collected from the brachial vein. After clotting at room temperature for 30 min, samples were centrifuged at 3,000 rpm (1,500 × g) for 15 min at 4 °C. Serum was promptly aliquoted into 1.5 mL microcentrifuge tubes and stored at −20 °C for subsequent analysis.

Serum inflammatory markers [interleukins (IL-1β, IL-6, IL-10), and tumor necrosis factor-α (TNF-α) were assayed with species-specific ELISA kits via a full-spectrum microplate reader (Multiskan GO, Thermo Scientific, USA). Concurrently, the serum oxidative markers malondialdehyde (MDA), glutathione peptide oxidase (GSH-Px), catalase (CAT), and total antioxidant capacity (T-AOC) were measured using a full-spectrum microplate reader with the corresponding kits.

ELISA kits for interleukin analysis [IL-1β (ml059835), IL-6 (ml059839), IL-10 (ml059830)], and TNF-α (ml002790) were sourced from Shanghai Mlbio Biotechnology Co., Ltd. (Shanghai, China). Oxidative parameters [MDA (G0109W), GSH-Px (G0204W), CAT (G0105W), and T-AOC (G0142W]) were obtained from Suzhou Grace Biotechnology Co., Ltd. (Jiangsu, China).

### 2.9 Indicators of gut health microbiota

Immediately post-slaughter, cecal contents were aseptically collected, flash-frozen in liquid nitrogen, and stored at −80 °C. Microbiome analysis was outsourced to Majorbio Bio-Pharm Technology Co., Ltd. (Shanghai, China), with amplification and sequencing procedures conducted according to the methodology of Nan et al. (2022). Briefly, microbial DNA was extracted from samples using a fecal DNA isolation kit. The V3-V4 hypervariable region of the bacterial 16S rRNA gene was amplified using primers 338F 51-ACTCCTACGGGAGGCAGCAG31) and 806R 51-GGACTACHVGGGTWTCTAAT31). PCR reactions were performed on an ABI GeneAmp^®^ 9,700 PCR system. The resulting amplicons were purified, pooled in equimolar ratios, and sequenced on an Illumina MiSeq PE300 platform (Illumina, San Diego, CA, USA) in paired-end mode according to the manufacturer's instructions. Data were processed and analyzed using the Majorbio Bio-Pharm Technology Co., Ltd. (Shanghai, China) Data Analysis Platform.

### 2.10 Statistical analysis

Spearman's rank correlation analysis was performed using the Majorbio Cloud Platform (www.majorbio.com). Correlation coefficients were calculated based on Spearman's distance metric and visualized in a correlation heatmap. Statistical significance was denoted as follows: *p* < 0.05 as “^*^,” *p* < 0.01 as “^**^” and *p* < 0.001 as “^***^.”

Experimental data were collated in Excel 2010 and subjected to statistical analysis using SPSS 20 (IBM, Armonk, NY, USA). A general linear model (GLM) univariate procedure was employed to evaluate the main effects of light intensity, cage position, and their interaction. When the interaction effect was not significant (*p* > 0.05), the main effects were interpreted directly. However, when a significant interaction (*p* < 0.05) was observed, the data were analyzed using One-Way ANOVA to assess the simple effects among the six treatment groups. Significant differences among groups were assessed by Duncan's multiple range test, with results expressed as mean and standard error of the mean (SEM). Statistical significance was defined as *p* < 0.05.

## 3 Results

### 3.1 Muscle quality

Light intensity had significant effects on breast muscle pH, meat color, cooking loss, and drip loss. The 4 lx treatment significantly improved pH_45min_, pH_24*h*_, and meat color (*p* < 0.05) while reducing cooking loss and drip loss (*p* < 0.05). Cage position had a significant effect on breast muscle shear force. Minimal shear force occurred in the middle cage position. Significant light × cage position interactions affected breast muscle quality indices, including pH_24*h*_ (*p* = 0.022), meat color (*p* = 0.014), protein content (*p* = 0.012), and intramuscular fat (IMF, *p* = 0.001). Specifically, the combined light cage variations changed the protein content by 1.39% (20.81% vs. 20.52%) and IMF by 26.75% (4.56% vs. 3.34%) (Table 3).

**Table 3:** Effect of light intensity and cage position on breast muscle quality in broilers.

**Items**	**pH_45min_**	**pH_24h_**	**Meat color**	**Shear force (N)**	**Cooking loss (%)**	**Drip loss (%)**	**Protein (%)**	**Moisture (%)**	**IMF^1^ (%)**
Ht	6.00	5.63^b^	67.94^a^	47.61	16.70	3.80	20.81^a^	70.04	3.36^b^
Hm	6.10	5.85^a^	72.50^a^	36.41	18.50	3.06	20.64^ab^	70.31	4.56^a^
Hs	6.07	5.86^a^	72.13^a^	52.07	18.26	3.21	20.72^a^	70.46	3.45^b^
Lt	5.67	5.51^c^	49.07^b^	63.65	22.14	6.63	20.67^ab^	69.94	3.77^ab^
Lm	5.68	5.59^bc^	32.51^c^	47.01	19.71	7.57	20.80^a^	70.36	3.34^b^
Ls	5.65	5.59^bc^	34.78^c^	48.94	21.68	7.31	20.52^b^	69.79	4.07^a^
SEM	0.02	0.01	1.54	2.47	0.58	0.25	0.03	0.15	0.13
**Light intensity**									
4 lx	6.06^a^	5.78^a^	70.85^a^	45.36	17.82^b^	3.36^b^	20.72	70.27	3.79
1.5 lx	5.67^b^	5.56^b^	38.79^b^	53.20	21.18^a^	7.17^a^	20.66	70.03	3.76
**Cage position**									
upper	5.83	5.57^b^	58.5	55.63^a	19.42	5.22	20.74	69.99	3.56
middle	5.89	5.72^a^	52.32	41.10^b	18.81	5.30	20.72	70.34	3.80
lower	5.86	5.73^a^	53.64	51.109^b	20.27	5.28	20.62	70.13	3.86
***p*** **-value**									
Light intensity	< 0.001	< 0.001	< 0.001	0.118	0.006	< 0.001	0.248	0.418	0.919
Cage position	0.249	< 0.001	0.235	0.075	0.829	0.988	0.142	0.635	0.480
Interaction	0.415	0.022	0.014	0.271	0.342	0.365	0.012	0.586	0.001

^*ab*^ In the same column, differences in lower case letters indicate a statistically significant difference (*p* < 0.05).

^1^IMF, intramuscular fat; SEM, standard error of the mean.

Light intensity had significant effects on leg muscle quality. The 4 lx treatment significantly improved pH_45min_, pH_24*h*_, meat color, and IMF of broiler leg muscle (*p* < 0.05), while reducing cooking loss, drip loss, and moisture (*p* < 0.05). Cage position had a significant effect on leg muscle pH_24*h*_, meat color, and cooking loss. Broiler leg muscle color was the highest in the middle cage position; pH_24*h*_ was significantly higher (*p* < 0.05) with decreasing cage position, and cooking loss tended to be lower. Significant light × cage position interactions affected pH_45min_ (*p* = 0.001), pH_24*h*_ (*p* < 0.001), and cooking loss (*p* = 0.023). Specifically, the Hm and Hs groups exhibited significantly higher pH_45min_ and pH_24*h*_ values compared to the other four groups (*p* < 0.05), while their cooking loss was significantly lower than that of the Ht, Lt, and Lm groups (*p* < 0.05) (Table 4).

**Table 4:** Effect of light intensity and cage position on leg muscle quality in broilers.

**Items**	**pH_45min_**	**pH_24h_**	**Meat color**	**Shear force (N)**	**Cooking loss (%)**	**Drip loss (%)**	**Protein (%)**	**Moisture (%)**	**IMF^1^ (%)**
Ht	6.24^b^	5.97^c^	60.66	30.87	25.98^a^	1.94	20.43	70.54	7.61
Hm	6.46^a^	6.19^b^	69.06	24.6	18.07^c^	1.69	20.39	70.32	8.40
Hs	6.51^a^	6.43^a^	66.71	23.63	19.21^c^	2.05	20.4	70.85	7.30
Lt	5.98^c^	5.89^c^	48.95	26.11	24.49^ab^	3.54	20.56	71.62	6.92
Lm	5.90^c^	5.86^c^	50.29	30.88	25.19^ab^	3.21	19.76	71.81	6.24
Ls	5.89^c^	5.87^c^	49.63	29.21	22.08^bc^	3.27	20.44	71.93	6.93
SEM	0.02	0.02	0.78	0.95	0.62	0.14	0.16	0.21	0.26
**Light intensity**									
4 lx	6.40^a^	6.20^a^	65.48^a^	26.37	21.09^b^	1.89^b^	20.35	70.41^b^	8.09^a^
1.5 lx	5.92^b^	5.87^b^	49.62^b^	28.73	23.59^a^	3.34^a^	20.27	71.92^a^	6.59^b^
**Cage position**									
upper	6.11	5.93^c^	54.81^b^	28.49	25.23^a^	2.74	20.53	71.37	7.04
middle	6.18	6.03^b^	59.67^a^	27.75	21.63^b^	2.45	20.01	70.78	7.71
lower	6.20	6.15^a^	58.17^ab^	26.42	20.14^b^	2.66	20.38	71.35	7.26
***p*** **-value**									
Light intensity	< 0.001	< 0.001	< 0.001	0.220	0.050	< 0.001	0.630	0.006	0.046
Cage position	0.146	< 0.001	0.041	0.671	0.005	0.683	0.522	0.779	0.945
Interaction	0.001	< 0.001	0.167	0.370	0.023	0.846	0.579	0.904	0.331

^*ab*^In the same column, differences in lower case letters indicate a statistically significant difference (*p* < 0.05).

### 3.2 Muscle amino acid composition

The total amino acid concentrations in the breast muscles showed no significant differences across the groups (*p* > 0.05, Supplementary Table 1), and individual adjustment of light intensity or cage position had no significant effect on amino acid content (*p* > 0.05). Significant light × cage position interactions affected Tyr levels (*p* = 0.045). Further analysis of inter-group variations revealed that the content of Tyr, an umami amino acid, was higher in the Hm and Lt groups, significantly higher than in the Ls group (*p* < 0.05) (Table 5).

**Table 5:** Effect of different light intensity and cage position on breast muscle amino acid in broilers (% total amino acids).

**Items**	**Tyr**	**EAA^1^**	**NEAA^2^**	**UAA^3^**
Ht	3.85^ab	45.43	54.57	45.75
Hm	4.16^a	45.55	54.45	44.87
Hs	4.08^ab	45.61	54.39	45.00
Lt	4.20^a	45.79	54.21	45.59
Lm	3.93^ab	45.69	54.31	45.01
Ls	3.57^b	48.18	51.82	43.79
SEM	0.07	0.33	0.33	0.32
**Light intensity**				
4 lx	4.03	45.53	54.47	45.21
1.5 lx	3.90	46.56	53.45	44.80
**Cage position**				
upper	4.03	45.61	54.39	45.67
middle	4.04	45.62	54.38	44.94
lower	3.82	46.90	53.11	44.40
***p*** **-value**				
Light intensity	0.329	0.134	0.134	0.525
Cage position	0.342	0.211	0.211	0.279
Interaction	0.045	0.271	0.271	0.668

^*ab*^ In the same column, differences in lower case letters indicate a statistically significant difference (*p* < 0.05).

^1^EAA: essential amino acids (Val+ Met+ Ile+ Leu+ Phe+ Lys+ His+ Thr).

^2^NEAA: nonessential amino acids (Tyr+ Asp+ Arg+ Pro+ Ser+ Glu+ Gly+ Ala).

^3^UAA: umami amino acids (Asp+ Glu+ Gly+ Ala+ Phe+ Tyr).

The total amino acid concentrations in the leg muscles showed no significant differences across the groups (*p* > 0.05, Supplementary Table 1). Light intensity predominantly affected Pro, with significantly elevated levels observed under 4 lx illumination (*p* < 0.05). Cage position primarily influenced Arg, with the highest concentration in the middle position, which significantly exceeded that in the lower position (*p* < 0.05). Significant light × cage position interactions affected Glu levels (*p* = 0.018). Further analysis of Glu showed no significant differences among Ht, Hm, Hs, and Lt groups (*p* > 0.05); Glu was highest in the Ls group, significantly higher than in the Lm group (*p* < 0.05) (Table 6).

**Table 6:** Effect of different light intensity and cage position on leg muscle amino acid in broilers (% total amino acids).

**Items**	**Leu**	**Tyr**	**Arg**	**Pro**	**Glu**	**Ala**	**EAA^1^**	**NEAA^2^**	**UAA^3^**
Ht	9.61	3.99	6.85	4.88	15.55^ab^	6.63	44.17	55.83	45.78
Hm	9.65	4.04	6.82	4.86	15.38^ab^	6.52	44.61	55.39	45.53
Hs	9.4	4.17	6.48	4.69	15.31^ab^	6.47	44.52	55.48	45.15
Lt	9.75	4.11	6.95	4.77	15.29^ab^	6.45	44.87	55.13	45.38
Lm	9.69	3.89	7.13	4.54	14.97^b^	6.98	44.48	55.52	45.67
Ls	9.41	4.15	6.50	4.30	16.11^a^	6.69	44.51	55.49	46.55
SEM	0.05	0.04	0.06	0.06	0.11	0.06	0.17	0.17	0.16
**Light intensity**									
4 lx	9.55	4.07	6.71	4.81^a	15.41	6.54	44.43	55.57	45.49
1.5 lx	9.62	4.05	6.86	4.54^b	15.46	6.71	44.62	55.38	45.87
**Cage position**									
upper	9.68	4.05	6.90^a^	4.83	15.42	6.54	44.52	55.48	45.58
middle	9.67	3.97	6.97^a^	4.70	15.18	6.75	44.54	55.46	45.60
lower	9.40	4.16	6.49^b^	4.50	15.71	6.58	44.52	55.48	45.85
***p*** **-value**									
Light intensity	0.532	0.818	0.233	0.035	0.850	0.179	0.598	0.598	0.244
Cage position	0.061	0.094	0.004	0.112	0.173	0.323	0.997	0.997	0.754
Interaction	0.857	0.292	0.586	0.635	0.018	0.111	0.581	0.581	0.079

^*ab*^ In the same column, differences in lower case letters indicate a statistically significant difference (*p* < 0.05).

^1^EAA: essential amino acids (Val+ Met+ Ile+ Leu+ Phe+ Lys+ His+ Thr).

^2^NEAA: nonessential amino acids (Tyr+ Asp+ Arg+ Pro+ Ser+ Glu+ Gly+ Ala).

^3^UAA: umami amino acids (Asp+ Glu+ Gly+ Ala+ Phe+ Tyr).

### 3.3 Muscle fatty acid composition

The total fatty acid concentrations in the breast muscles showed no significant differences across the groups (*p* > 0.05, Supplementary Table 1). Light intensity had no significant effect on fatty acid composition or breast muscle content (*p* > 0.05). However, regarding cage position, the concentrations of C16:0 and *trans-*C18:1 in the middle position increased significantly (*p* < 0.05) compared with those in the upper and lower positions, whereas those of *cis-*C18:1 decreased significantly (*p* < 0.05). A significant light × cage position interaction was observed exclusively for C16:0 (*p* < 0.001). Further analysis of C16:0 showed no significant differences among Ht, Hm, Lm, and Ls groups (*p* > 0.05); however, the concentrations in these groups were significantly higher than in the Hs and Lt groups (*p* < 0.05) (Table 7).

**Table 7:** Effect of different light intensity and cage position on breast muscle fatty acid in broilers (% total fatty acids).

**Items**	**C16:0**	**C18:0**	**trans-C18:1**	**cis-C18:1**
Ht	30.09^a^	9.41	15.01	9.69
Hm	29.79^a^	9.36	15.01	9.32
Hs	27.76^b^	11.34	13.30	11.06
Lt	27.91^b^	10.23	14.47	11.25
Lm	29.66^a^	9.76	15.78	9.75
Ls	29.29^a^	9.99	13.39	11.16
SEM	0.12	0.19	0.36	0.17
**Light intensity**				
4 lx	29.21	10.04	14.439	10.02
1.5 lx	28.96	10.00	14.55	10.72
**Cage position**				
upper	29.00^b^	9.82^ab^	14.74^ab^	10.47^a^
middle	29.73^a^	9.56^b^	15.40^a^	9.53^b^
lower	28.52^b^	10.67^a^	13.34^b^	11.11^a^
***p*** **-value**				
Light intensity	0.288	0.912	0.884	0.06
Cage position	0.004	0.085	0.103	0.008
Interaction	< 0.001	0.083	0.766	0.214
**Items**	**SFA** ^1^	**MUFA** ^2^	**PUFA** ^3^	**PUFA/SFA**	**n-3PUFA** ^4^	**n-6PUFA** ^5^	**n-6/n-3**
Ht	46.22	29.87	23.91	0.52	1.27	23.24	18.30
Hm	44.43	30.29	25.28	0.57	1.17	24.71	21.81
Hs	47.13	28.64	24.22	0.51	1.27	23.55	18.43
Lt	45.08	30.7	24.21	0.55	1.05	23.72	22.89
Lm	44.88	31.12	24.00	0.52	1.06	23.49	22.63
Ls	46.02	29.49	24.48	0.57	1.16	23.93	20.93
SEM	0.61	0.48	0.26	0.01	0.04	0.27	0.69
**Light intensity**							
4 lx	45.93	29.60	24.47	0.53	1.14	23.83	20.18
1.5 lx	45.33	30.44	24.23	0.54	1.02	23.71	22.82
**Cage position**							
upper	45.65	30.29	24.06	0.53	1.14	23.48	20.09
middle	44.66	30.70	24.64	0.55	1.04	24.10	22.22
lower	46.58	29.07	24.36	0.53	1.12	23.74	20.18
***p*** **-value**							
Light intensity	0.630	0.400	0.652	0.880	0.531	0.829	0.240
Cage position	0.457	0.382	0.666	0.614	0.797	0.656	0.576
Interaction	0.832	0.966	0.390	0.530	0.529	0.395	0.596

^*ab*^ In the same column, differences in lower case letters indicate a statistically significant difference (*p* < 0.05).

^1^SFA: saturated fatty acids (C10:0 + C12:0 + C14:0 + C15:0 + C16:0 + C17:0 + C18:0 + C20:0 + C23:0).

^2^MUFA: monounsaturated fatty acids (C14:1 + C16:1 + trans-C18:1 + cis-C18:1 + C20:1).

^3^PUFA: polyunsaturated fatty acids (C18:2n6 + C18:3n3 + C20:2n6 + C20:3n6).

^4^n-3 PUFA: C18:3n3 + C20:5n3.

^5^n-6 PUFA:C18:2n6 + C20:2n6 + C20:3n6.

The total fatty acid concentrations in the leg muscles showed no significant differences across the groups (*p* > 0.05, Supplementary Table 1). Compared to 1.5 lx, the 4 lx treatment reduced n-3 polyunsaturated fatty acids (PUFA) and *cis-*C18:1 (*p* < 0.05) and increased SFA and the n-6/n-3 PUFA ratio (*p* < 0.05). Upper position SFA concentrations were higher than lower position (*p* < 0.05), but statistically similar to those in the middle position (*p* > 0.05), primarily driven by parallel increases in C10:0, C12:0, and C13:0. Conversely, n-3 PUFA levels decreased in the upper vs. the lower positions (*p* < 0.05), with no difference between the upper and middle levels (*p* > 0.05). Significant light × cage position interactions were observed for C13:0 (*p* = 0.038), C15:0 (*p* = 0.037), C20:0 (*p* = 0.038), and SFA (*p* = 0.005). Further analysis revealed that C13:0 and C20:0 concentrations in the Ht group were statistically similar to those in the Hm group but significantly higher than those in the other four groups (*p* < 0.05). For C15:0, the Ht and Hm groups showed no significant differences compared to the Lt and Lm groups (*p* > 0.05), yet were significantly higher than the Ls group (*p* < 0.05). Regarding SFA, the Ht and Hm groups did not differ significantly from the Lt group (*p* > 0.05) but were significantly higher than those in the Lm and Ls groups (*p* < 0.05) (Table 8).

**Table 8:** Effect of different light intensity and cage position on leg muscle fatty acid in broilers (% total fatty acids).

**Items**	**C10:0**	**C12:0**	**C13:0**	**C15:0**	**cis-C18:1**	**C18:3n3**	**C20:0**
Ht	0.02	0.07	0.05^a^	0.11^a^	9.33	0.40	0.31^a^
Hm	0.01	0.06	0.04^ab^	0.11^a^	8.96	0.43	0.29^ab^
Hs	0.01	0.05	0.02^c^	0.08^b^	10.67	0.53	0.14^c^
Lt	0.01	0.05	0.03^bc^	0.09^ab^	10.18	0.48	0.20^bc^
Lm	0.01	0.05	0.02^c^	0.10^ab^	9.77	0.56	0.14^c^
Ls	0.01	0.05	0.03^c^	0.10^ab^	10.49	0.51	0.18^c^
SEM	< 0.01	< 0.01	< 0.01	< 0.01	0.10	0.01	0.01
**Light intensity**							
4 lx	0.02	0.06	0.04^a^	0.10	9.66^b	0.46^b^	0.25^a^
1.5 lx	0.01	0.05	0.03^b^	0.09	10.15^a	0.52^a^	0.17^b^
**Cage position**							
upper	0.02^a^	0.06^a^	0.04^a^	0.10	9.75^b	0.44^b^	0.26^a^
middle	0.01^ab^	0.06^ab^	0.03^ab^	0.10	9.37^b	0.49^ab^	0.22^ab^
lower	0.01^b^	0.05^b^	0.02^b^	0.09	10.58^a	0.52^a^	0.16^b^
***p*** **-value**							
Light intensity	0.141	0.038	0.015	0.217	0.025	0.030	0.016
Cage position	0.033	0.060	0.032	0.137	0.001	0.079	0.029
Interaction	0.085	0.060	0.038	0.037	0.082	0.100	0.038
**Items**	**SFA** ^1^	**MUFA** ^2^	**PUFA** ^3^	**PUFA/SFA**	**n-3PUFA** ^4^	**n-6PUFA** ^5^	**n-6/n-3**
Ht	42.49^a^	31.33	26.17	0.62	0.90	25.77	28.94
Hm	42.41^a^	30.64	26.94	0.64	0.93	26.51	28.68
Hs	38.22^b^	34.29	27.49	0.72	1.13	26.96	23.38
Lt	40.30^ab^	33.15	26.54	0.65	1.04	26.06	25.40
Lm	38.37^b^	33.66	27.96	0.71	1.16	27.4	23.02
Ls	40.06^b^	32.4	27.54	0.69	1.05	27.03	25.51
SEM	0.29	0.45	0.40	0.01	0.01	0.39	0.40
**Light intensity**							
4 lx	41.04^a^	32.09	26.87	0.66	1.06^b	26.41	26.00^a^
1.5 lx	39.58^b^	33.07	27.35	0.69	1.12^a	26.83	24.51^b^
**Cage position**							
upper	41.40^a^	32.24	26.36	0.64	0.94^b	25.92	25.17
middle	40.40^ab^	32.15	27.45	0.68	1.09^ab	26.96	24.65
lower	39.14^b^	33.34	27.51	0.70	1.12^a	26.99	24.44
***p*** **-value**							
Light intensity	0.028	0.299	0.557	0.170	0.039	0.606	0.039
Cage position	0.027	0.509	0.436	0.108	0.079	0.470	0.188
Interaction	0.005	0.111	0.882	0.140	0.130	0.909	0.129

^*ab*^ In the same column, differences in lower case letters indicate a statistically significant difference (*p* < 0.05).

^1^SFA: saturated fatty acids (C10:0 + C12:0 + C13:0 + C14:0 + C15:0 + C16:0 + C17:0 + C18:0 + C20:0 + C23:0).

^2^MUFA: monounsaturated fatty acids (C14:1 + C16:1 + C17:1 + trans-C18:1 + cis-C18:1 + C20:1).

^3^PUFA: polyunsaturated fatty acids (C18:2n6 + C18:3n6 + C18:3n3 + C20:2n6 + C20:3n6). ^4^n-3 PUFA: C18:3n3 + C20:5n3. ^5^n-6 PUFA: C18:2n6 + C18:3n6 + C20:2n6 + C20:3n6.

### 3.4 Serum inflammatory and oxidative markers

Light intensity had significant effects on T-AOC, with the 4 lx treatment significantly increasing T-AOC (*p* < 0.05). Cage position had a significant effect on IL-10, TNF-α, and MDA. The middle cage position exhibited significantly higher IL-10 levels than those in the upper cage position (*p* < 0.05) while demonstrating the lowest TNF-α and MDA concentrations. Significant interaction effects between light intensity and cage position were observed for serum IL-1β, IL-6, TNF-α, and MDA levels (*p* < 0.01). Notably, the Hm group exhibited lower serum IL-1β and MDA levels than both the Ht and Hs groups. Similarly, the Lm group showed reduced IL-1β and MDA levels compared with the Lt and Ls groups, whereas the Hm group achieved the lowest levels of both IL-1β and MDA across all experimental groups (*p* < 0.05) (Table 9).

**Table 9:** Effects of light intensity and cage position on inflammatory and oxidative in broilers.

**Items**	**IL-1β (pg/mL)**	**IL-6 (pg/mL)**	**IL-10 (pg/mL)**	**TNF-α (pg/mL)**	**MDA (nmol/ml)**	**GSH-Px (U/ml)**	**CAT (U/ml)**	**T-AOC (nmol/ml)**
Ht	100.53^a^	25.48^a	39.37^bc^	46.21^c^	0.77^b^	301.42	33.52^a^	656.61
Hm	71.34^b^	27.61^a	45.20^bc^	63.35^b^	0.43^c^	316.86	26.67^ab^	658.81
Hs	99.28^a^	11.72^c	46.30^bc^	67.88^ab^	0.47^bc^	295.27	24.30^b^	663.44
Lt	101.24^a^	19.91^b	35.06^c^	78.63^a^	0.84^b^	317.02	28.54^ab^	650.91
Lm	76.74^b^	18.19^b	50.25^b^	48.33^c^	0.81^b^	329.55	27.88^ab^	646.02
Ls	96.33^a^	29.96^a	63.45^a^	69.94^ab^	1.18^a^	332.60	33.15^a^	643.44
SEM	2.62	0.62	1.71	1.62	0.05	12.3	1.78	13.01
**Light intensity**							
4 lx	93.72	21.60	43.62	59.15	0.56	304.52	28.15	659.67^a^
1.5 lx	88.10	22.64	49.59	65.63	0.66	326.39	29.86	646.32^b^
**Cage position**							
upper	100.64	22.69	37.22^b^	62.42^ab^	0.79^ab^	309.22	31.03	653.23
middle	71.29	22.83	47.73^a^	55.84^b^	0.66^b^	323.2	27.27	652.98
lower	97.81	20.84	54.87^a^	68.91^a^	0.80^a^	330.93	28.73	653.02
***p*** **-value**							
Light intensity	0.290	0.412	0.090	0.053	0.557	0.121	0.055	0.030
Cage position	0.101	0.358	0.001	0.009	0.004	0.650	0.114	0.764
Interaction	< 0.001	< 0.001	0.049	< 0.001	< 0.001	0.576	0.040	0.429

^*ab*^ In the same column, differences in lower case letters indicate a statistically significant difference (*p* < 0.05).

### 3.5 Cecal microbiota composition

#### 3.5.1 Venn diagrams, microbial community composition, and principal component analysis

**Figure 1:**
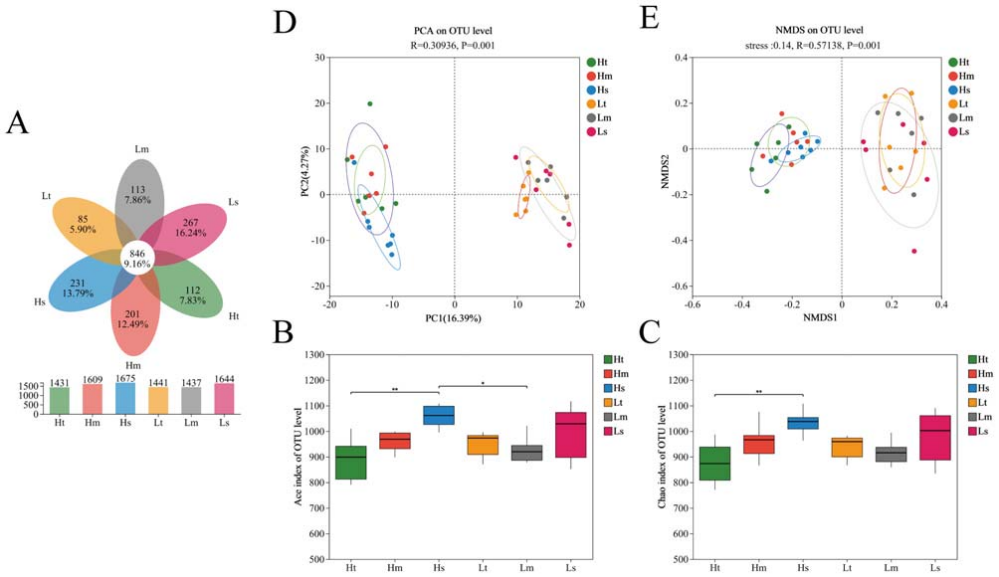
Light intensities and cage positions on the microbial community composition and principal component analysis of broiler cecum. **(A)** Venn diagram of OTUs in gut microbiota. **(B)** ACE index and **(C)** Chao1 index showing intergroup differences test in gut microbiota α-diversity. **(D)** PCA and **(E)** NMDS analyses based on Bray–Curtis distance of bacterial communities; n = 7.

The Venn diagram illustrates the shared and unique operational taxonomic units (OTUs) across the sample groups (Figure 1A). Cecal microbiota analysis identified 9,237 OTUs per group, with 846 OTUs (9.16% of the total) shared universally. In addition, it can be seen that the total number of OTUs unique to the 4 lx treatment was greater than that under the 1.5 lx treatment. However, the difference in the number of unique OTUs caused by the cage position was greater, and the number of unique OTUs caused by the cage position under both light conditions was in the order of upper < middle < lower.

Alpha diversity metrics revealed significant variations in microbial community richness (Figures 1B, C). The Hs group exhibited substantially higher ACE (*p* < 0.01) and Chao1 (*p* < 0.01) indices than the Ht group. ACE levels also differed significantly between the Hs and Lm groups (*p* < 0.05). Beta diversity analyses (PCA and NMDS; Figures 1D, E) demonstrated distinct clustering patterns (PCA: R = 0.309, *p* = 0.001; NMDS: Stress = 0.14, R = 0.571, *p* = 0.001), with clear segregation of microbial communities between the 4 lx and 1.5 lx treatments, indicating that the treatment significantly altered the gut microbiota beta-diversity."

#### 3.5.2 Taxonomic analysis

**Figure 2:**
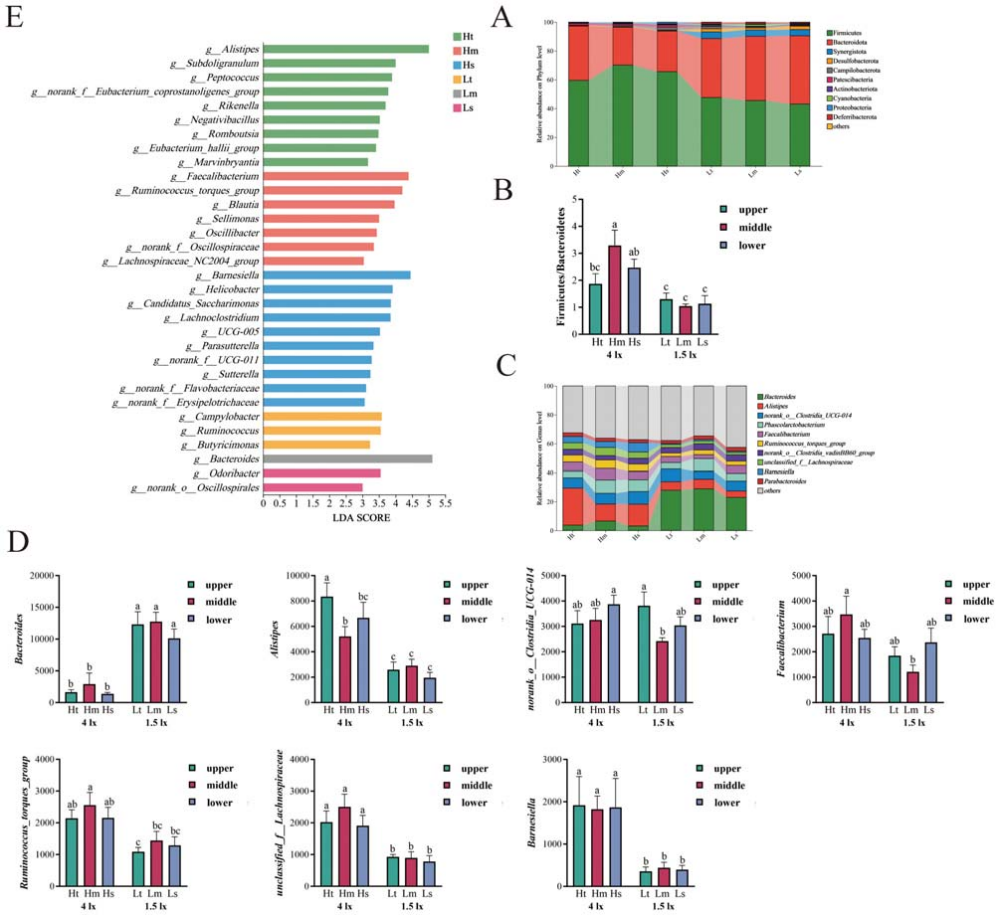
Effects of different light intensities and cage positions on the main differential bacteria in the microbial community of broiler cecum. **(A)** Relative abundance of gut microbiota at the phylum level. **(B)** Proportion of Firmicutes and Bacteroidetes. **(C)** Relative abundance of gut microbiota at the genus level. **(D)** Differential bacteria at the genus level, and **(E)** histogram of LDA value distribution; labels denote P < 0.05; values are presented as mean ± SEM; n = 7.

Taxonomic analysis of the top 10 phyla, including Firmicutes, Bacteroidetes, Desulfobacterota, Campylobacterota, Patescibacteria, Cyanobacteria, and Proteobacteria, showed significant intergroup variation (Figure 2A). Firmicutes and Bacteroidetes dominated microbial profiles. Light intensity exerted a stronger influence than the cage position. Firmicutes abundance was higher under the 1.5 lx treatment than under the 4 lx treatment, whereas Bacteroidetes abundance was lower under the 1.5 lx treatment. The highest Firmicutes/Bacteroidetes (F/B) ratio was found in the Hm group under the synergistic effect of light and caging, which was significantly higher than that of the Ht and 1.5 lx treatment groups (*p* < 0.05), and the lowest F/B was found in the Lm group (Figure 2B).

Further analysis of the genus-level cecal microbial composition revealed that the main differentiated bacteria detected in the six groups were *Bacteroides, Alistipes, norank_Clostridia_UCG-014, Faecalibacterium, Ruminococcus_torques_group, unclassified_f_ Lachnospiraceae, Barnesiella* (Figure 2C). From the point of view of light intensity, at 4 lx treatment, *Alistipes* accounted for more than 11.81% and was the most dominant genus in all groups, at 1.5 lx, *Bacteroides* was the most dominant genus in all groups (>22.96%). *Bacteroides* abundance was significantly lower (*p* < 0.05), whereas *Barnesiella* abundance was significantly higher (*p* < 0.05) under the 4 lx treatment than under the 1.5 lx treatment, and *Alistipes* was significantly higher (*p* < 0.05) in the Ht and Hm group. Concurrently, *Faecalibacterium, Ruminococcus_torques_group*, and *unclassified_f_Ruminococcaceae* were the most abundant in the Hm group. Among these, *Faecalibacterium* was significantly higher in the Hm group than in the Lm group (*p* < 0.05) and was not significantly different from the other groups (*p* > 0.05). The *Ruminococcus_torques_group* and *unclassified_f_Lachnospiraceae* groups in the 4 lx treatment were significantly higher than those in the 1.5 lx treatment (*p* > 0.05) (Figure 2D).

The LEfSe algorithm is illustrated in Figure 2E. A total of 32 genus-level bacteria differed among cecal contents. However, there were only six classifications with LDA scores >4. These included *Alistipes* (Ht), *Subdoligranulum* (Ht), *Faecalibacterium* (Hm), *Ruminococcus_torques_group* (Hm), *Barnesiella* (Hs) in the 4 lx treatment and *Bacteroides* (Lm) in the 1.5 lx. The most influential bacteria with LDA Scores greater than five were *Alistipes* in the Ht group and *Bacteroide* in the Lm group.

#### 3.5.3 Spearman's correlation

**Figure 3:**
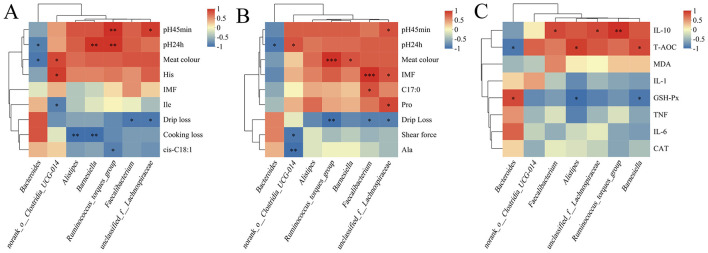
Correlation of differential bacteria with serum biochemical indices, meat quality, muscle fatty acids, and muscle amino acids. **(A)** Spearman correlation analysis of differential intestinal bacteria (genus level) with breast meat quality, differential fatty acids, and amino acids. **(B)** Spearman correlation analysis of differential intestinal bacteria (genus level) with leg meat quality, differential fatty acids, and amino acids. **(C)** Spearman correlation analysis of differential intestinal bacteria (genus level) with inflammatory and oxidative stress factors.

Spearman's correlation analysis revealed significant associations between cecal microbiota, serum inflammatory/oxidative markers, and meat quality parameters (Figure 3). *Alistipes* and *Barnesiella* showed significant positive correlations with the antioxidant marker T-AOC, whereas both genera were negatively correlated with cooking loss in breast muscle. Additionally, *Barnesiella* positively correlated with leg muscle color and negatively correlated with drip loss. *Faecalibacterium, Ruminococcus_torques_group*, and *unclassified_f_Lachnospiraceae* were positively correlated with the inflammatory factor IL-10 while simultaneously demonstrating negative correlations with drip loss in both breast and leg muscles. Moreover, *Ruminococcus_torques_group* and *unclassified_f_Lachnospiraceae* were positively correlated with breast muscle pH_45min_, with *Ruminococcus_torques_group* also showing a positive correlation with leg muscle color. Notably, *Faecalibacterium* and *unclassified_f_Lachnospiraceae* were positively associated with IMF content in the leg muscle.

## 4 Discussion

In this study, analysis of both light intensity and cage position revealed that broilers subjected to 4 lx lighting and middle-tier placement exhibited higher IMF content, higher pH values, and improved meat color, along with reduced cooking and drip loss. Furthermore, 16S rRNA analysis indicated that both light intensity and cage position altered the gut microbiota composition. These microbial shifts paralleled the observed variations in meat quality as well as systemic inflammatory and oxidative status.

Alterations in muscle nutritional composition are closely associated with meat quality, with IMF content being a key factor. Parameters such as pH, meat color, cooking loss, and drip loss are critical indicators for evaluating meat quality (Li et al., 2023; Kucha et al., 2021). In the current study, we observed that broilers exposed to 4 lx light intensity and in the middle cage position exhibited higher IMF content from the perspective of light and cage tiers. Further comprehensive analysis revealed that both breast and leg muscles in the Hm group had the highest pH values, superior meat color, and reduced cooking and drip losses. This may be attributed to the more uniform lighting and comfortable cage environment in the Hm group, which provided greater environmental stability and reduced the risk of chronic stress, thereby creating more favorable conditions for broiler development Xu et al. (2024) demonstrated that ChREBP-mediated upregulation of Them1 responds to chronic stress induced by environmental changes, thereby reducing stress-induced glycolysis. Consequently, the enhanced environmental stability in the Hm group may have alleviated chronic stress, potentially reducing stress-induced glycolysis and lactic acid accumulation, thus slowing pH decline. A rapid decrease in pH is often associated with pale, soft, and exudative (PSE) meat defects (Matarneh et al., 2021). These mechanisms align with the observed outcomes in the Hm group, including higher pH, improved meat color, and reduced cooking and drip losses, confirming that Hm was the optimal treatment in this experiment.

Amino acid composition significantly influences muscle flavor, with flavor amino acids, particularly the aromatic amino acid Tyr, serving as key precursors for the formation of phenolic compounds that contribute to muscle flavor (Fu et al., 2024). In the present study, the EAA content across all muscle samples ranged from 44.16% to 48.18%, which is consistent with the previously reported range of 30%−50% in chicken meat (Zhou et al., 2024; Liu et al., 2023), indicating good agreement with existing literature. Tyr was identified as differentially abundant amino acids in breast muscle, whereas Glu showed significant differences in leg muscles, with higher levels observed in the Ht and Hm groups. Notably, Tyr and Glu are flavor amino acids known to enhance meat flavor. Tyr, which belongs to the aromatic amino acid group, has been reported to generate volatile flavor compounds, such as phenols and benzenoids via microbial or transaminase pathways, which are critical for flavor development (Deng et al., 2024; Fu et al., 2024). Furthermore, Pro is believed to influence IMF content and water-holding capacity (as reflected in drip and cooking losses), with higher concentrations generally indicating superior muscle quality (Zhou et al., 2024; Wang and Maeda, 2018). Therefore, the elevated levels of these amino acids in the Ht and Hm groups suggested that broilers reared under higher light intensities and in the middle cage position may exhibit improved meat quality.

Fatty acids, as key components of IMF, are important indicators for evaluating muscle quality (Yu et al., 2023). In the present study, when analyzed separately by light intensity and cage tier, breast muscle from broilers reared under 4 lx light and in the middle cage position exhibited reduced desaturation of C16:0, accompanied by decreased endogenous synthesis of C16:1. Concurrently, enhanced desaturation of C18:0 was observed along with increased endogenous synthesis of trans-C18:1. Consistent with our findings, Realini et al. (2021) reported that increased IMF was associated with greater desaturation of C18:0 and a lower C16:1/C16:0 ratio. This agreement supports the view that breast muscle from the Hm group, characterized by higher levels of C16:0 and trans-C18:1 and lower levels of C16:1 and C18:0, may be of superior quality. No significant differences were detected in the overall fatty acid categories of the breast muscle, indicating that while light and cage tiers mildly affected certain individual fatty acids, they did not significantly alter the overall fatty acid profile. In leg muscles, the Ht and Hm groups showed higher levels of SFA, driven primarily by parallel increases in C10:0, C12:0, and C13:0. Previous studies have suggested that C10:0 and C12:0 may alleviate inflammation by inhibiting NF-κB activation and MAP phosphorylation (Huang et al., 2014). Moreover, Zhan et al. (2024) explicitly demonstrated that C12:0 levels significantly influenced the antioxidant capacity. Additionally, when evaluated by light intensity and Cage position separately, reduced n-3 PUFA levels were observed in leg muscle under 4 lx light and middle cage conditions, whereas the 4 lx treatment led to an increased n-6/n-3 PUFA ratio. Since elevated n-6 PUFA levels may promote inflammatory responses and increase pro-inflammatory cytokine levels (Weng et al., 2025; Kang et al., 2024), subsequent analyses were conducted to assess anti-inflammatory and antioxidant statuses.

Superior meat quality stems from a favorable physiological status, particularly improved anti-inflammatory and antioxidant capacities, which are closely associated with enhanced meat quality (Cao et al., 2021). In the present study, when analyzed by light intensity and Cage position separately, broilers under 4 lx light exhibited higher T-AOC, whereas those in the middle cage position showed significantly higher IL-10 levels than the upper tier group, along with lower concentrations of TNF-α and MDA. Further comprehensive analysis revealed that the Hm group demonstrated concurrently lower IL-1β and MDA levels, as well as higher IL-10 and T-AOC. Previous studies have indicated that IL-1β and TNF-α are biomarkers of a pro-inflammatory state, while IL-10 may function to limit and ultimately terminate inflammatory responses (Han et al., 2016; Voshagh et al., 2024). MDA is a product of lipid peroxidation, and T-AOC serves as a comprehensive indicator of antioxidant status (Cao et al., 2014). The concurrent reduction in IL-1β and MDA and elevation of IL-10 and T-AOC in the Hm group indicate a better systemic inflammatory and oxidative status. Substantial evidence suggests that inflammatory and oxidative states are closely linked to muscle protein metabolism and lipid oxidation and that reducing the inflammatory burden and oxidative damage can improve meat quality (Cao et al., 2021; Crossland et al., 2019; Son et al., 2022). For instance, Crossland et al. (2019) proposed that inflammatory status influences muscle protein turnover, thereby affecting meat quality. Similarly, Son et al. (2022) demonstrated that alleviating oxidative stress reduces cooking loss and shear force, thereby enhancing meat quality, which is consistent with the lower cooking loss and shear force observed in the Hm group in our study. Based on these findings, we speculate that the regulation of meat quality by light intensity and Cage positionis associated with the modulation of systemic inflammatory and oxidative statuses. It should be noted that oxidative and inflammatory markers were measured in serum rather than muscle tissue. While systemic status often influences tissue physiology, future studies measuring muscle-specific markers (e.g., muscle ROS levels or local cytokine expression) are needed to directly verify the muscle-level mechanisms.

Light intensity and cage position influence the microbial community structure, and previous studies suggest that such microbial shifts may be linked to systemic anti-inflammatory and antioxidant status (Zhao et al., 2024). Based on the 16S rRNA sequencing analysis, we observed distinct compositional differences in the gut microbiota among the treatment groups. When the effects of light intensity and Cage position were comprehensively analyzed, the Hm group exhibited the highest F/B ratio. Previous studies have suggested that an increased F/B ratio is associated with enhanced fat deposition (Hu et al., 2019). In our study, the Hm group also demonstrated higher IMF content, suggesting a possible connection between these two factors. We acknowledge, however, that the F/B ratio is a simplified metric with inherent variability. It reflects gross changes in community structure but does not capture the full complexity of microbial metabolic functions. Thus, although the elevated F/B ratio aligns with the increased IMF content in our study, we hypothesize that it serves as a broad indicator of dysbiosis or metabolic shift, likely driven by the proliferation of specific metabolically relevant Firmicutes genera, rather than a simplistic causal mechanism. At the genus level, we analyzed the top 10 most abundant bacterial genera and identified seven differentially abundant genera by filtering. These included *Bacteroides, Alistipes*, and *Barnesiella* from Bacteroidetes as well as *norank_f__Clostridia_UCG-014, Faecalibacterium, Ruminococcus_torques_group*, and *unclassified_f__Lachnospiraceae* from Firmicutes. Numerous studies have indicated that improvements in meat quality are often accompanied by structural changes in gut microbiota. Su et al. reported that *Bacteroides, Alistipes*, and *unclassified_f__Lachnospiraceae* participate in the PPAR signaling pathway, regulating lipid metabolism in broilers, and thereby influencing muscle pH, meat color, shear force, and cooking loss (Su et al., 2024). In another study by Ruan et al., blended oil diets that affected meat color and drip loss also led to differences in the abundance of *Ruminococcus_torques_group* among groups (Ruan et al., 2024). Consistent with these findings, in the present study, both light intensity and cage position concurrently influenced both meat quality and microbial composition.

Spearman's correlation analysis further indicated potential associations between specific genera and meat quality traits. *Alistipes* and *Barnesiella* were significantly negatively correlated with cooking loss in breast muscle. *Barnesiella* was also positively correlated with meat color and negatively correlated with drip loss in the leg muscles. Meanwhile, *Faecalibacterium, unclassified_f__Lachnospiraceae*, and *Ruminococcus_torques_group* showed significant negative correlations with drip loss in both the breast and leg muscles. Most of these genera, which are positively associated with superior meat quality, have been reported to possess anti-inflammatory or antioxidant functions in other contexts. For example, *Alistipes* is linked to the regulation of inflammation and oxidation (Wang et al., 2020; Huang et al., 2024), and studies such as those by Song et al. (2023) have indicated that *Barnesiella* can stimulate immune cells and alleviate inflammation. In our study, both genera were significantly and positively correlated with T-AOC. Additionally, *Faecalibacterium* and *unclassified_f__Lachnospiraceae*, belonging to the phylum Bacillota, are known to produce butyrate and enhance intestinal barrier function, confirming their beneficial roles (Miquel et al., 2013). Lyu et al. (2021) further suggested that *Ruminococcus_torques_group* promotes fat deposition. In the current study, *Faecalibacterium, unclassified_f__Lachnospiraceae*, and *Ruminococcus_torques_group* were also significantly positively correlated with the anti-inflammatory cytokine IL-10. Taken together, these results indicated that the Hm group exhibited an increased abundance of bacteria putatively involved in the regulation of systemic inflammatory and oxidative status. The enrichment of beneficial genera such as *Alistipes* and *Faecalibacterium* may contribute to an enhanced anti-inflammatory and antioxidant capacity, as suggested by their correlations with IL-10 and T-AOC. We speculate that this microbial profile could establish a physiological environment conducive to metabolic stability in muscle tissue, which may largely explain the observed slower decline in pH, stabilized meat color, increased IMF content, and reduced cooking and drip loss.

## 5 Conclusions

The present study indicates that compared to the 1.5 lx treatment (groups Lt, Lm, and Ls), the 4 lx treatment (groups Ht, Hm, and Hs) significantly increased muscle pH, improved meat color, and reduced both cooking loss and drip loss. Broilers in the middle position exhibited the lowest breast meat shear force and the highest leg meat color. Furthermore, leg meat pH_24*h*_ significantly increased as the cage tier decreased, while cooking loss showed a decreasing trend. Among all the groups, Hm exhibited the most favorable meat quality outcomes. Broilers in the middle position exhibited the lowest breast meat shear force and the highest leg meat color. Furthermore, leg meat pH_24*h*_ significantly increased as the cage tier decreased, while cooking loss showed a decreasing trend. Notably, beneficial bacterial genera, such as *Alistipes* and *Faecalibacterium*, were notably enriched in the Hm group, which collectively coincided with an improved systemic inflammatory and oxidative status. These findings suggest that the enhanced anti-inflammatory and antioxidant capacities may be linked to the observed microbial shifts. In conclusion, the interaction between light intensity and cage position optimized meat quality, a process accompanied by distinct changes in gut microbiota structure, inflammatory and oxidative responses.

## Data Availability

The data presented in the study are deposited in the NCBI Sequence Read Archive (SRA) repository, accession number PRJNA1423903.
