# Supplementary material for: Light intensity and cage position affect meat quality by regulating intestinal flora, inflammation and oxidation in broilers
Source: Front Microbiol. 2026 Feb 25;17:1729385. doi: 10.3389/fmicb.2026.1729385 (PMC12975879; doi:10.3389/fmicb.2026.1729385)
Supplement: Supplementary file 1 [file Table_1.docx]

Supplementary Material

# Supplementary Tables

**TABLE S1.** Effect of different light intensity and cage position on total fatty acid and amino acid in broilers.

| **Items** | **Breast muscle** | | **Leg muscle** | |
| --- | --- | --- | --- | --- |
|  | **TFA (mg/100g freeze-dried meat)^1^** | **TAA(g/100g fresh meat)^2^** | **TFA(mg/100g freeze-dried meat)** | **TAA (g/100g fresh meat)** |
| Ht | 1.46 | 20.75 | 3.40 | 21.64 |
| Hm | 1.15 | 20.16 | 3.46 | 19.58 |
| Hs | 1.31 | 20.99 | 4.11 | 19.58 |
| Lt | 1.18 | 22.38 | 3.54 | 20.54 |
| Lm | 1.53 | 20.99 | 4.08 | 20.43 |
| Ls | 2.13 | 21.38 | 3.46 | 21.41 |
| SEM | 0.13 | 0.54 | 0.10 | 0.38 |
| Light intensity | | | | |
| 4 lx | 1.31 | 20.63 | 3.65 | 20.27 |
| 1.5 lx | 1.61 | 21.58 | 3.69 | 20.79 |
| Cage position |  |  |  |  |
| upper | 1.32 | 21.57 | 3.47 | 21.09 |
| middle | 1.34 | 20.58 | 3.77 | 20 |
| lower | 1.72 | 21.18 | 3.78 | 20.49 |
| P-value |  |  |  |  |
| Light intensity | 0.25 | 0.38 | 0.834 | 0.495 |
| Cage position | 0.39 | 0.751 | 0.368 | 0.512 |
| Interaction | 0.25 | 0.892 | 0.060 | 0.293 |

In the same column, differences in lower case letters indicate a statistically significant difference (P < 0.05). 1 TFA: total fatty acids; 2 TAA: total amino acids.

**TABLE S2 Effect of different light intensity and cage position on breast muscle amino acid in broilers (% total amino acids)**

| **Items** | **Val** | **Met** | **Leu** | **Phe** | **Thr** | **Asp** | **Arg** | **Ser** | **Glu** | **Gly** | **Ala** | **Ile** | **Lys** | **His** | **Pro** |
| --- | --- | --- | --- | --- | --- | --- | --- | --- | --- | --- | --- | --- | --- | --- | --- |
| Ht | 4.99 | 2.59 | 9.05 | 5.5 | 4.41 | 9.74 | 6.65 | 3.62 | 15.33 | 4.85 | 6.49 | 5.25 | 9.40 | 4.23 | 4.05 |
| Hm | 5.04 | 2.75 | 9.39 | 4.83 | 4.58 | 9.73 | 6.74 | 3.64 | 14.88 | 4.65 | 6.63 | 5.26 | 9.30 | 4.41 | 4.04 |
| Hs | 5.06 | 2.66 | 9.25 | 5.18 | 4.48 | 9.58 | 6.57 | 3.6 | 15 | 4.61 | 6.55 | 5.15 | 9.14 | 4.67 | 4.40 |
| Lt | 5.051 | 2.819 | 9.18 | 5.78 | 4.44 | 9.58 | 6.58 | 3.64 | 15.03 | 4.54 | 6.47 | 5.08 | 9.15 | 4.30 | 4.17 |
| Lm | 5.03 | 2.77 | 9.36 | 5.39 | 4.47 | 9.59 | 6.89 | 3.49 | 14.92 | 4.59 | 6.58 | 5.32 | 9.30 | 4.04 | 4.31 |
| Ls | 5.33 | 2.99 | 9.64 | 5.72 | 4.69 | 11.08 | 6.71 | 3.68 | 12.66 | 4.5 | 6.26 | 5.53 | 10.37 | 3.93 | 3.35 |
| SEM | 0.06 | 0.06 | 0.13 | 0.16 | 0.05 | 0.24 | 0.08 | 0.04 | 0.42 | 0.05 | 0.07 | 0.05 | 0.14 | 0.09 | 0.13 |
| Light intensity |  |  |  |  |  |  |  |  |  |  |  |  |  |  |  |
| 4 lx | 5.03 | 2.67 | 9.23 | 5.17 | 4.49 | 9.68 | 6.65 | 3.62 | 15.07 | 4.7 | 6.55 | 5.22 | 9.28 | 4.44 | 4.17 |
| 1.5 lx | 5.14 | 2.86 | 9.39 | 5.63 | 4.53 | 10.08 | 6.73 | 3.61 | 14.21 | 4.55 | 6.44 | 5.31 | 9.61 | 4.09 | 3.94 |
| Cage position |  |  |  |  |  |  |  |  |  |  |  |  |  |  |  |
| upper | 5.02 | 2.71 | 9.12 | 5.64 | 4.43 | 9.66 | 6.61 | 3.63 | 15.18 | 4.69 | 6.48 | 5.17 | 9.28 | 4.26 | 4.11 |
| middle | 5.03 | 2.76 | 9.38 | 5.11 | 4.53 | 9.66 | 6.81 | 3.56 | 14.9 | 4.62 | 6.61 | 5.29 | 9.30 | 4.22 | 4.18 |
| lower | 5.19 | 2.83 | 9.44 | 5.45 | 4.58 | 10.33 | 6.64 | 3.64 | 13.83 | 4.56 | 6.41 | 5.34 | 9.76 | 4.30 | 3.88 |
| P-value |  |  |  |  |  |  |  |  |  |  |  |  |  |  |  |
| Light intensity | 0.359 | 0.142 | 0.543 | 0.154 | 0.635 | 0.413 | 0.632 | 0.875 | 0.315 | 0.132 | 0.387 | 0.345 | 0.265 | 0.052 | 0.393 |
| Cage position | 0.398 | 0.744 | 0.566 | 0.383 | 0.416 | 0.435 | 0.506 | 0.692 | 0.4 | 0.539 | 0.46 | 0.326 | 0.313 | 0.929 | 0.606 |
| Interaction | 0.617 | 0.621 | 0.805 | 0.917 | 0.404 | 0.292 | 0.793 | 0.496 | 0.468 | 0.551 | 0.661 | 0.084 | 0.095 | 0.173 | 0.087 |

**TABLE S3 Effect of different light intensity and cage position on leg muscle amino acid in broilers (% total amino acids)**

| **Items** | **Val** | **Met** | **Ile** | **Phe** | **Lys** | **His** | **Thr** | **Asp** | **Ser** | **Gly** | **Leu** | **Tyr** | **Ala** |
| --- | --- | --- | --- | --- | --- | --- | --- | --- | --- | --- | --- | --- | --- |
| Ht | 4.57 | 2.69 | 5.07 | 5 | 9.95 | 3 | 4.28 | 9.5 | 3.32 | 5.12 | 9.61 | 3.99 | 6.63 |
| Hm | 4.67 | 2.7 | 5.13 | 5.12 | 10.1 | 3.01 | 4.25 | 9.54 | 3.29 | 4.93 | 9.65 | 4.04 | 6.52 |
| Hs | 4.65 | 2.67 | 4.95 | 5.07 | 9.75 | 3.22 | 4.18 | 9.39 | 3.32 | 4.73 | 9.4 | 4.17 | 6.47 |
| Lt | 4.68 | 2.87 | 5.18 | 5.23 | 9.8 | 3.05 | 4.3 | 9.54 | 3.27 | 4.77 | 9.75 | 4.11 | 6.45 |
| Lm | 4.66 | 2.91 | 5.31 | 4.91 | 10.09 | 2.81 | 4.09 | 9.6 | 3.09 | 5.31 | 9.69 | 3.89 | 6.98 |
| Ls | 4.65 | 2.88 | 5.04 | 5.29 | 10.09 | 3 | 4.15 | 9.54 | 3.43 | 4.77 | 9.41 | 4.15 | 6.69 |
| SEM | 0.03 | 0.07 | 0.05 | 0.08 | 0.13 | 0.06 | 0.04 | 0.05 | 0.06 | 0.11 | 0.05 | 0.04 | 0.06 |
| Light intensity |  |  |  |  |  |  |  |  |  |  |  |  |  |
| 4 lx | 4.63 | 2.69 | 5.05 | 5.06 | 9.93 | 3.08 | 4.24 | 9.48 | 3.31 | 4.93 | 9.55 | 4.07 | 6.54 |
| 1.5 lx | 4.66 | 2.89 | 5.18 | 5.14 | 10 | 2.96 | 4.18 | 9.56 | 3.26 | 4.95 | 9.62 | 4.05 | 6.71 |
| Cage position |  |  |  |  |  |  |  |  |  |  |  |  |  |
| upper | 4.63 | 2.78 | 5.13 | 5.2 | 9.88 | 3.03 | 4.29 | 9.52 | 3.29 | 4.95 | 9.68 | 4.05 | 6.54 |
| middle | 4.66 | 2.8 | 5.22 | 5.01 | 10.1 | 2.91 | 4.17 | 9.57 | 3.19 | 5.12 | 9.67 | 3.97 | 6.75 |
| lower | 4.65 | 2.78 | 5 | 5.18 | 9.92 | 3.11 | 4.17 | 9.47 | 3.38 | 4.75 | 9.40 | 4.16 | 6.58 |
| *P*-value |  |  |  |  |  |  |  |  |  |  |  |  |  |
| Light intensity | 0.578 | 0.178 | 0.178 | 0.605 | 0.807 | 0.351 | 0.545 | 0.363 | 0.692 | 0.916 | 0.532 | 0.818 | 0.179 |
| Cage position | 0.881 | 0.985 | 0.164 | 0.67 | 0.761 | 0.442 | 0.478 | 0.622 | 0.404 | 0.412 | 0.061 | 0.094 | 0.323 |
| Interaction | 0.752 | 0.998 | 0.914 | 0.423 | 0.718 | 0.622 | 0.716 | 0.864 | 0.529 | 0.424 | 0.857 | 0.292 | 0.111 |

**TABLE S4 Effect of different light intensity and cage position on breast muscle fatty acid in broilers (% total fatty acids)**

| **Items** | **C10:0** | **C12:0** | **C14:0** | **C14:1** | **C15:0** | **C16:1** | **C17:0** | **C18:2n6** | **C18:3n3** | **C20:0** | **C20:1** | **C20:2n6** | **C20:3n6** | **C23:0** | **C20:5n3** |
| --- | --- | --- | --- | --- | --- | --- | --- | --- | --- | --- | --- | --- | --- | --- | --- |
| Ht | 0.03 | 0.16 | 0.77 | 0.29 | 0.25 | 4.2 | 0.4 | 21.46 | 0.67 | 0.94 | 0.67 | 0.77 | 1.01 | 4.17 | 0.54 |
| Hm | 0.03 | 0.11 | 0.78 | 0.25 | 0.2 | 5.16 | 0.32 | 23.3 | 0.57 | 0.7 | 0.54 | 0.61 | 0.8 | 3.14 | 0.54 |
| Hs | 0.03 | 0.15 | 0.75 | 0.27 | 0.25 | 3.33 | 0.41 | 21.63 | 0.67 | 0.95 | 0.68 | 0.85 | 1.07 | 5.49 | 0.54 |
| Lt | 0.03 | 0.1 | 0.74 | 0.22 | 0.18 | 4.28 | 0.3 | 22.32 | 0.5 | 0.61 | 0.49 | 0.6 | 0.79 | 4.97 | 0.55 |
| Lm | 0.02 | 0.1 | 0.82 | 0.22 | 0.17 | 4.9 | 0.28 | 22.22 | 0.51 | 0.59 | 0.48 | 0.61 | 0.67 | 3.47 | 0.55 |
| Ls | 0.02 | 0.12 | 0.8 | 0.24 | 0.2 | 4.18 | 0.32 | 22.41 | 0.56 | 0.69 | 0.52 | 0.65 | 0.86 | 4.59 | 0.55 |
| SEM | 0.01 | 0.01 | 0.02 | 0.01 | 0.02 | 0.25 | 0.03 | 0.36 | 0.04 | 0.07 | 0.04 | 0.06 | 0.07 | 0.35 | 0.03 |
| Light intensity | | | | | | | | | | | | | | |  |
| 4 lx | 0.03 | 0.14 | 0.77 | 0.27 | 0.23 | 4.23 | 0.37 | 22.13 | 0.64 | 0.86 | 0.63 | 0.74 | 0.96 | 4.27 | 0.54 |
| 1.5 lx | 0.03 | 0.11 | 0.79 | 0.22 | 0.18 | 4.45 | 0.3 | 22.32 | 0.52 | 0.63 | 0.5 | 0.62 | 0.78 | 4.35 | 0.55 |
| Cage position | | | | | | | | | | | | | | |  |
| upper | 0.03 | 0.13 | 0.75 | 0.25 | 0.22 | 4.24 | 0.35 | 21.89 | 0.58 | 0.78 | 0.58 | 0.68 | 0.9 | 4.57 | 0.54 |
| middle | 0.03 | 0.11 | 0.8 | 0.24 | 0.19 | 5.03 | 0.3 | 22.76 | 0.54 | 0.65 | 0.51 | 0.61 | 0.73 | 3.31 | 0.55 |
| lower | 0.03 | 0.14 | 0.77 | 0.26 | 0.23 | 3.76 | 0.36 | 22.02 | 0.62 | 0.82 | 0.6 | 0.75 | 0.97 | 5.04 | 0.54 |
| P-value |  |  |  |  |  |  |  |  |  |  |  |  |  |  |  |
| Light intensity | 0.237 | 0.122 | 0.617 | 0.101 | 0.161 | 0.66 | 0.184 | 0.797 | 0.131 | 0.143 | 0.116 | 0.286 | 0.188 | 0.915 | 0.921 |
| Cage position | 0.347 | 0.616 | 0.656 | 0.799 | 0.590 | 0.143 | 0.576 | 0.576 | 0.697 | 0.604 | 0.623 | 0.587 | 0.362 | 0.148 | 0.826 |
| Interaction | 0.669 | 0.734 | 0.708 | 0.823 | 0.833 | 0.648 | 0.886 | 0.471 | 0.829 | 0.826 | 0.812 | 0.752 | 0.956 | 0.60 | 0.242 |

**TABLE S5 Effect of different light intensity and cage position on leg muscle fatty acid in broilers (% total fatty acids)**

| **Items** | **C14:0** | **C14:1** | **C16:0** | **C16:1** | **C17:0** | **C17:1** | **C18:0** | **trans-C18:1** | **C18:2n6** | **C18:3n6** | **C20:1** | **C20:2n6** | **C20:3n6** | **C23:0** | **C20:5n3** |
| --- | --- | --- | --- | --- | --- | --- | --- | --- | --- | --- | --- | --- | --- | --- | --- |
| Ht | 0.82 | 0.18 | 27.82 | 5.66 | 0.19 | 0.14 | 9.94 | 15.68 | 24.56 | 0.25 | 0.34 | 0.52 | 0.43 | 3.17 | 0.54 |
| Hm | 0.81 | 0.2 | 26.58 | 6.67 | 0.18 | 0.12 | 10.33 | 14.35 | 25.18 | 0.25 | 0.33 | 0.64 | 0.44 | 4.00 | 0.56 |
| Hs | 0.89 | 0.21 | 25.30 | 7.17 | 0.16 | 0.10 | 9.05 | 15.79 | 26.19 | 0.17 | 0.35 | 0.33 | 0.27 | 2.53 | 0.58 |
| Lt | 0.87 | 0.19 | 26.02 | 6.69 | 0.16 | 0.10 | 9.86 | 15.63 | 25.01 | 0.21 | 0.36 | 0.5 | 0.34 | 3.00 | 0.56 |
| Lm | 0.91 | 0.19 | 25.27 | 7.08 | 0.15 | 0.07 | 9.03 | 16.16 | 25.81 | 0.21 | 0.38 | 1.03 | 0.35 | 2.75 | 0.59 |
| Ls | 0.93 | 0.19 | 25.75 | 6.45 | 0.17 | 0.07 | 9.45 | 14.86 | 26.07 | 0.19 | 0.34 | 0.4 | 0.38 | 3.41 | 0.58 |
| SEM | 0.02 | 0.01 | 0.26 | 0.28 | 0.01 | 0.01 | 0.21 | 0.26 | 0.32 | 0.01 | 0.01 | 0.1 | 0.02 | 0.23 | 0.05 |
| Light intensity | | | | | | | | | | | | | | |  |
| 4 lx | 0.84 | 0.22 | 26.57 | 6.5 | 0.18 | 0.12 | 9.77 | 15.28 | 25.31 | 0.22 | 0.34 | 0.5 | 0.38 | 3.23 | 0.56 |
| 1.5 lx | 0.9 | 0.19 | 25.66 | 6.74 | 0.16 | 0.08 | 9.45 | 15.55 | 25.63 | 0.21 | 0.36 | 0.64 | 0.36 | 3.05 | 0.58 |
| Cage position | | | | | | | | | | | | | | |  |
| upper | 0.84 | 0.19 | 26.92 | 6.17 | 0.17 | 0.12 | 9.9 | 15.66 | 24.79 | 0.23 | 0.35 | 0.51 | 0.38 | 3.09 | 0.55 |
| middle | 0.86 | 0.2 | 25.9 | 6.88 | 0.17 | 0.1 | 9.68 | 15.26 | 25.49 | 0.23 | 0.35 | 0.84 | 0.4 | 3.37 | 0.58 |
| lower | 0.91 | 0.2 | 25.52 | 6.81 | 0.16 | 0.09 | 9.25 | 15.32 | 26.13 | 0.18 | 0.35 | 0.36 | 0.32 | 2.97 | 0.58 |
| P-value |  |  |  |  |  |  |  |  |  |  |  |  |  |  |  |
| Light intensity | 0.065 | 0.9 | 0.101 | 0.672 | 0.094 | 0.051 | 0.442 | 0.603 | 0.632 | 0.418 | 0.261 | 0.491 | 0.511 | 0.698 | 0.167 |
| Cage position | 0.254 | 0.786 | 0.108 | 0.539 | 0.572 | 0.256 | 0.449 | 0.795 | 0.276 | 0.077 | 0.972 | 0.196 | 0.347 | 0.757 | 0.153 |
| Interaction | 0.673 | 0.807 | 0.203 | 0.451 | 0.069 | 0.809 | 0.268 | 0.124 | 0.887 | 0.445 | 0.316 | 0.702 | 0.137 | 0.199 | 0.176 |
